# Patient satisfaction after removal of locking plates in proximal humeral fractures – worth the surgery under functional and cosmetic aspects?

**DOI:** 10.1016/j.jseint.2024.04.010

**Published:** 2024-04-27

**Authors:** Ludwig Jägerhuber, Georg Siebenbürger, Evi Fleischhacker, Fabian Gibert, Wolfgang Böcker, Tobias Helfen

**Affiliations:** Department of Orthopaedics and Trauma Surgery, LMU University Hospital, LMU Munich, Germany

**Keywords:** Proximal humeral fracture, Locked plating, Functional outcome, Constant Score, Cosmetics, Scar, Implant removal

## Abstract

**Background:**

Locking plates are one of the most frequently used implants in surgical treatment of displaced proximal humeral fractures. In spite of this established implant and a standardized approach, reduced shoulder function might remain a mid-to long-term issue, furthermore scars may influence patient satisfaction as a cosmetic issue. Indications for a second surgery to remove implant and/or scar revision are common questions in this context.

The aim of the present study was to assess the benefit of a second surgery, including implant removal and scar revision surgery, on patients’ satisfaction under functional and cosmetic aspects.

**Methods:**

Patients following displaced proximal humeral fractures treated by open reduction and internal fixation with a locking plate osteosynthesis via a delto-pectoral approach following implant removal were included retrospectively. A follow-up took place anamnestically before the incident or the primary surgery [A], before second surgery [B], and after second surgery (scar revision/implant removal) [C]. Functional outcome (Constant Score (CS)) of both shoulders was obtained using a patient-reported outcome measure to evaluate the contralateral side as well as percentage CS (%CS). Furthermore, the cosmetic outcome was evaluated for color, contour, and size.

**Results:**

The clinical data of n = 81 patients with displaced proximal humeral fractures and the consecutive open reduction and internal fixation with a locking plate (51 women = 63.0% and 30 men = 37.0%; mean age: 53.7 ± 16.6 years) via a standardized deltopectoral approach could be included. The mean CS) before sustaining the humeral fracture or primary surgery [A] was anamnestically 85.8 ± 8.5 points and %CS 99.4 ± 8.4%. After open reduction and internal with a locking plate osteosynthesis [B], the mean CS was 72.2 ± 9.1 points and %CS 84.5 ± 8.7%. Following the scheduled implant removal and scar revision [C], the CS was 80 ± 13.1 points and %CS 92.3 ± 14.1%. After primary surgery, 26 patients (32.1%) complained about the scar because of color, contour, or size before the second surgery, and 23 patients (28.4%) afterwards.

**Conclusion:**

Implant removal after locking plate osteosynthesis in proximal humeral fractures via a delto-pectoral approach leads to an improved outcome both functionally and cosmetically. CS as well as scar situation and patients’ overall satisfaction could be improved with a second surgery. Nevertheless, the need for a second surgery depends on the patients’ functional and cosmetic demands.

Proximal humeral fractures are among the most common fractures, accounting for about 6% of all fractures. Women are being affected more often than men, and the mean age being reported accounts for 65 years.^6^ In elderly patients, the most common accident mechanism is a low-speed trauma with a fall from a standing position, whereas younger patients are typically sustaining proximal humeral fractures associated with sports and motorcycle accidents.^7^^,^^8^,^19^ The therapeutic ambition is to recover a functional outcome like before the accident accompanied by patient satisfaction. Fracture type, age, bone quality, functional demand, and comorbidity have to be taken into account for the individual suspect. Approximately 80% of all humeral fractures are minimal or nondisplaced and can therefore be treated conservatively with a good functional result.^26^ 20% of humeral fractures indicate for surgical treatment because of displacement according to the modified Neer criteria.^25^ At present, one of the most frequently used implants for surgical treatment of proximal humeral fractures is the locking plate. Up to now, there is no clear evidence for superiority of any surgical treatment in literature (plate vs. nail vs. arthroplasty.^12^^,^^14^^,^^23^, ^24^, ^25^^,^^28^

Not uncommonly, proximal humeral fractures are associated with long-term complaints, such as discomfort or movement-depending pain as well as reduced shoulder function irrespective of bony consolidation.^16^^,^^27^ Mechanical outlet impingement in combination with plate and subacromial space is the most important factor.^10^^,^^18^^,^^21^ This leads to a loss of more than 50% of the shoulder function in specific cases.^30^ The Constant Score (CS) as one of the most frequent applied tools for the assessment of the functional shoulder outcome, shows a protracted recovering process with a significant improvement after months.^21^ Especially in physically demanding professions, this led to longer inability to work.^9^

Loss of shoulder function over a longer period represents a persistent topic in the literature. Adhesions, as well as implant-associated or mechanical aspects, are under discussion.^13^^,^^17^ Acklin et al found improvements in shoulder function following implant removal in a small collective of n = 20 cases.^1^ Hirschmann et al showed a significant recovery of shoulder function in terms of mobility in all planes, strength, and reduction in pain intensity in the first year after surgery, but stagnation to an inferior level after 1 year, evenafter the implant was removed.^15^ Arthrolysis in cases of posttraumatic shoulder stiffness might be an additional procedure to improve the functional outcome.^20^ Nevertheless, no general recommendation for implant removal has been made so far due to an additional surgery including perioperative risks and complications.^11^^,^^22^

Skin closure and scar management can be another significant contributor to patients’ satisfaction. Scars remain a stigmatizing visible reminder of the surgery. Tethered scars are a common surgical problem that may produce contour defects, lead to functional restriction, or cause other abnormalities.^29^ Implant removal seems a suitable moment for scar revision, which might influence the satisfaction and outcome additionally. The overall proportion of patient satisfaction regarding scars is currently not supported by any clear evidence.

Assuming function and cosmesis are crucial for the patient after surgically treated proximal humeral fractures, the aim of the present study was to assess the impact of a second implant removal surgery on patients’ satisfaction in these aspects. The authors hypothesized a positive effect of implant removal on function and cosmesis.

## Methods

The study design is in accordance with the recommendations of the Declaration of Helsinki and was approved by the Ethical Committee of the Medical University of Munich (#20-1083).

### Patient recruitment

Patients with displaced proximal humeral fractures treated surgically by locking plate osteosynthesis that underwent implant removal were identified in an in-house patient database retrospectively. The inclusion criteria for the study were patient age of at least 18 years, written informed consent, and the presence of a displaced proximal humeral fracture with subsequent locking plate osteosynthesis. The monocentric study was performed at the Level 1 trauma center of Munich University Hospital using the in-hospital IT system (SAP) as a data source. The following parameters were extracted from the IT system, see Table I. The minimum follow-up was defined as 12 months after implant removal.

**Table I:** Parameters in the IT system that were obtained before starting the study.

Date of birth	Age	Date of surgery	Date of injury
Time between injury and operation	Fracture type AO/OTA-classification	Implant at stay	Follow-up time
Injured side	Type of implant		

*AO/OTA*, “Arbeitsgemeinschaft für Osteosynthesefragen” Foundation/Orthopaedic Trauma Association.

### Intervention

All fractures were initially immobilized by a Gilchrist bandage by the trauma surgeon on-duty in the emergency department. Operative treatment was performed to in-house standard. Including implants were locking plate osteosynthesis (either PHILOS, Depuy-Synthes, Raynham, MA, USA or Humeral SuturePlate, Arthrex, Naples, FL, USA) via a delto-pectoral approach.^2^ All study investigators were very experienced with both devices and have completed their learning curves. The performing clinic has a large shoulder center with a high level of case numbers, surgical training programs, and standardization of shoulder surgeries from the surgical approach up to after-treatment protocol. Implant removal was performed by a shoulder surgeon (≥50 shoulder surgeries per year) in a standard manner; moreover, a mobilization of the shoulder under anesthesia was performed in every case.

### Postoperative rehabilitation

Postoperative rehabilitation followed an internal evidence-based guideline. All patients received physiotherapy beginning on day 1 after surgery (primary and secondary surgery). Restrictions on range of motion were based on the following scheme: flexion/extension 60/0/0° and internal/external rotation 20/0/20° in the first two weeks. From the third week on, flexion was allowed up to 80° and rotation increased by 10°. From week 5, flexion up to 120° was possible, and from week 7, after X-ray control and radiological consolidation, pain-adapted full mobilization was performed.

### Follow-up

Patients were presented with a self-assessment questionnaire for bilateral shoulder function as well as scar situation and cosmetic satisfaction. Attached hereto, they were asked to fill out 3 self-assessments: before trauma/first surgery [A], 6 months before secondary surgery (implant removal and scar revision) [B], and 12 months after secondary surgery (implant removal and scar revision) [C]. In order to measure the shoulder function, the modified CS was used for every point of assessment in this study. This score assigns the subjective parameters of pain, daily function, the objective range of motion, and strength for the glenohumeral joint.^6^^,^^5^ The pain is indicated using a numerical analog scale with values from 0 to 10. The mobility of the shoulder joint in all spatial planes is presented using sample images, and the appropriate degree of mobility is selected by the patient. The strength of the arm is measured by the patients themselves using different weights. Furthermore, the CS of the uninjured side (cCS) as well as the CS as a percentage (%CS) of the injured side to the uninjured side was acquired. At the second study aspect, patients were asked to provide information on the scar situation, such as scar length and subjective assessment. Scar length was measured using a measuring tape. All other aspects (color, contour, and size) were subjective values that the patients individually assessed. Possible complications and disturbing factors like color, contour, and size were enquired. Additionally, a final school mark was given with regard to the cosmesis and satisfaction of the scar. At the 3^rd^ date of survey, a final assessment was conducted to check whether the removal surgery has been worthwhile.

### Power calculation and statistical evaluation

The primary outcome parameter was the Constant-Murley Score (0-100 points). In a case or power calculation for unpaired samples and steady targets, an effect size of 5 points difference at a standard deviation of 10 points was expected. These values were taken from established, published original articles for both proceedings, checked for plausibility, and implemented. From here the parameters were as follows: delta = 15, standard deviation= 15, alpha = 0.05, power = 0.8, resulting in n = 63 cases. To safeguard the quality of the study, the dropout rate should not exceed 20% (13 cases). Therefore, a final cohort size of n = 76 was calculated.

Continuous variables were described by means and standard deviation and were compared using t-test, one-way ANOVA, and Tukey HSD. Normality assumption was evaluated based on Shapiro-Wilk test. Categorical variables were analyzed using the Chi-Square test. The level of significance for all tests was set at *P* < .05. Statistical analysis was performed using SPSS (IBM SPSS Statistics for Windows, version 24.0 Released 2016; IBM Corp., Armonk, NY, USA).

## Results

Clinical data of 81 patients with displaced proximal humeral fractures and an angular stable locking plate (51 women ≙ 63.0% and 30 men ≙ 37.0%; mean age: 53.7 ± 16.6 years) were available for this study. The affected side was in 49 patients (60.5%) the left shoulder, and in 32 patients, (39.5%) the right shoulder. The fracture patterns according to the “Arbeitsgemeinschaft für Osteosynthesefragen” Foundation/Orthopaedic Trauma Association classification, andfracture diversification was as follows: AO11-A1 6 patients (7.4%), AO11-A2 4 patients (4.9%), AO11-A3 13 patients (16%), AO11-B1 16 patients (19.8%), AO11-B2 14 patients (17.3%), AO11-B3 3 patients (17.3%), AO11-C1 9 patients (11.1%), AO11-C2 8 patients (9.9%), and AO11C3 8 patients (9.9%). See Table II.

**Table II:** Fracture type distribution of the included collective according to the AO/OTA classification.

Fracture type: AO/OTA	Cases [n(%)]
11- A1	6 (7.4)
11- A2	4 (4.9)
11- A3	13 (16.0)
11- B1	16 (19.7)
11- B2	14 (17.2)
11- B3	3 (3.7)
11- C1	9 (11.1)
11- C2	8 (9.8)
11- C3	8 (9.8)

*AO/OTA*, “Arbeitsgemeinschaft für Osteosynthesefragen” Foundation/Orthopaedic Trauma Association.

### Functional outcome

For detailed functional outcome see Table III. Of n = 81 patients treated by locking plate osteosynthesis for displaced proximal humeral fractures, the mean CS before the fracture, at [A], was 85.8 ± 8.5 points, the CS of the uninjured contralateral shoulder (cCS) was 86.2 ± 6.6 points and the %CS 99.4 ± 8,4%. At [B] 13.2 ± 5.3 months after primary surgery, the mean CS was 72.2 ± 9.1 points, the CS of the uninjured contralateral shoulder (cCS) was 85.6 ± 7.2 and the %CS 84.5 ± 8.7%. At [C] the CS was 80 ± 13.1 points, the CS of the uninjured contralateral side was 86.9 ± 11.3 points and the %CS 92.3 ± 14.1%. (CS[A] vs. CS[B]: *P* = 1; CS[A] vs. CS[C] *P* < .01; CS[B] vs. CS[C] *P* < .01; cCS[A] vs. cCS[B] *P* = .9; cCS[A] vs. cCS[C] *P* = .9; cCS[B] vs. cCS[C] *P* = .7; %CS[A] vs. %CS[B] *P* < .01; %CS[A] vs. %CS[C] *P* = .01; %CS[B] vs. %CS[C] *P* = .03). ANOVA analysis showed statistical significant differences between the CS before the fracture and after implant removal and between CS after surgery and after implant removal, no significant differences for cCS at [A], [B] or [C] but for %CS between the values before the injury, after locked plating and after implant removal. Mean implant lifetime was 13.2 ± 5.3 months. A detailed testing for A/B/C fracture classification and CS before and after angular stable plating and implant removal showed no significant difference in the three fracture pattern types according to AO. (Detailed *P* values are therefore not defined here.)

**Table III:** Functional outcome before trauma [A], after locking plate osteosynthesis [B] and after implant removal [C].

Time	CS f (MW ± STD)	CS u (MW ± STD)	Difference between CS f und CS u
[A]	85.8 ± 8.5	86.5 ± 6.6	0.6%
[B]	72.2 ± 9.1	85.6 ± 7.2	15.5%
[C]	80 ± 13.1	86.9 ± 11.3	7.7%

*CS f*, fractured side; *CS u*, uninjured side; *MW*, mean value; *STD*, standard deviation.

### Cosmesis

The mean scar length of the delto-pectoral approach was 10.0 ± 2.8 cm at [B] and 10.3 ± 2.9 cm at [C] (*P* = .9). In no case, additional surgical approaches or surgery associated to scars were necessary. At [B], n = 27, patients (32.3%) suffered because of color, contour, and size of the scar. In n = 3 (3.7%) cases, patients were unsatisfied about the color of the scar, n = 12 (14.8%) because of the size, and n = 9 (11.1%) because of contouring. One patient (1.2%) suffered from color and size, as well as one patient (1.2%) from size and contouring of the scar. In n = 54 (66.7%) cases, patients were overall satisfied with the color, size, and contouring of the scar. At [C], n = 25 (30.9%) suffered after implant removal because of color, contour, and size of the scar. After implant removal, n = 2 patients (2.5%) were unsatisfied about the color of the scar, n = 12 patients (14.8%) because of the size, and n = 6 (7.4%) because of contouring. n = 3 patients suffered from size and contouring of the scar (3.7%) and n = 1 because of color, size, and contour (1.2%). n = 56 (69.1%) patients were overall satisfied with the color, size, and contour of the scar for comparison at [C]. For detailed testing of cosmetic results see Table IV. Chi-square testing showed therefore no statistically significant difference between both groups before and after implant removal. All patients rated the final outcome after implant removal with a mark 2.2 ± 1.1 equaling “B” in an “A” to “F” US marking system. When asked whether implant removal was worthwhile, 92% of patients answered in the affirmative.

**Table IV:** Detailed cosmetic results: time after locking plate osteosynthesis [B] and after implant removal [C].

Cosmetic deficit	Patients (n) [B]	%/total [B]	Patients (n) [C]	% [C]	*P* value
Total	26	32.1	23	28.4	.82
Color	3	3.7	2	2.5	.65
Dimension	12	14.8	12	14.8	1
Contour	9	11.1	6	7.4	.41
Color and contour	1	1.2	0	0	-
Dimension and contour	1	1.2	3	3.7	.31
Color, dimension, and contour	0	3.7	1	1.2	-
None of the above	54	66.7	56	69.1	.73

## Discussion

The primary objective of this study was to assess and differentiate functional and cosmetic outcomes following implantation and subsequent removal of locking plates in patients with proximal humeral fractures. The observed collective shows a mean age of 53 years. This is below the typical age distribution of around 66 years described in the literature. At 63% female, our collective is below the described gender distribution of approximately 72% female for proximal humerus fractures. In this regard, our observed cohort aligns with the existing literature, but it includes younger patients and more men than the typical cohort in the literature with locking plate osteosynthesis of a displaced proximal humeral fracture. In our cohort, the fracture type according to the AO classification showed no significant outcome difference in shoulder function. The cohort included a heterogenous distribution of fracture pattern. However, this has to be critically considered under the aspect of the insufficiently large collective in each fracture severity of the AO classification (n < 33). As a limitation, data on handedness was not collected. Also, no more anamnesis determination was performed at the B or C follow-up.

Prior to the occurrence of trauma, majority of participants had no significant deficits of shoulder function in both shoulders. Even about 1 year after surgery, a reduced shoulder function with resulting dissatisfaction was reported. These findings 12 months after implantation are comparable to the literature.^22^ The impact of cosmetic aspects after plate implantation was low threshold in the present collective. Scar appearance, length, and color were assessed as adequate after the first surgery.

The main indication for implant removal in the present collective was reported and measured functional limitations. Implant removal led to significant increase of shoulder function and overall satisfaction. The mobility of the shoulder clearly approached the origin function after implant removal and should therefore be regarded as the main indicator for implant removal. An increase in satisfaction due to mostly cosmetic aspects could not be perceived, even when considering the already good satisfaction before implant removal.

Patients could clearly reflect their reasons for an implant removal desire. Functional goals dominated cosmetical issues. Thus, a consensus of indication on the part of the patients and surgeons could be monitored. Under these conditions, it was possible to achieve a remarkable satisfaction of all cases performing the implant removal surgery. Therefore, the authors recommend critical evaluation of the indication in patients with primarily cosmetic aspects as a less appropriated collective.

The risk profile of the nonessential surgical procedure of implant removal proved to be unproblematic in the present study. Complications such as infection, refracture, intraoperative issues, or new-onset scar proliferation have not been observed. The second surgery also did not result in any enlargement of the scars or worsening of the scar aspect in any case.

The present study is subject to several limitations that warrant discussion. Firstly, the study design was retrospective. The CS was used for shoulder function in this study on the basis of established questionnaires in the working group. Other scores such as the standardized index of shoulder function or the shoulder function index were not used due to the length that would otherwise result and for reasons of clarity of the questionnaires. Additionally, the heterogenous distribution of fracture types as well as n = 11 performing surgeons might be initially inconsistent circumstances. Nevertheless, the performing clinic has a large shoulder center with a high level of case numbers, surgical training programs, and standardization of shoulder surgeries from the surgical approach up to after-treatment protocol. Implant removal was performed by a shoulder surgeon (≥50 shoulder surgeries/year) in a standard manner; moreover, a mobilization of the shoulder under anesthesia was performed in every case. Despite the various possible approaches, the results of this study are only applicable to the delto-pectoral approach. In this study, the CS was determined by the patients' own assessment based on the questionnaire modified from original publication. This method has already been approved in several other studies and is considered to be equivalent to the survey carried out by an examiner.^3^^,^^4^

## Conclusion

The findings of this study demonstrate that treatment with a locking plate osteosynthesis for displaced proximal humeral fractures leads to a good functional outcome and satisfactory cosmesis. However, despite successful surgical treatment and subsequent aftercare, patients may still experience medium-term functional deficits and cosmetic concerns, which can impact their overall satisfaction following locking plate osteosynthesis of the proximal humerus through a delto-pectoral approach. Although there may be a temporary decline in shoulder function following the fracture and initial surgery, patients tend to achieve functional recovery after implant removal. This study aimed to evaluate the impact of these complaints on the decision-making process of implant removal as well as the influence of satisfaction to treatment conclusion. The present study could clearly show the shoulder function as indicator and predictor for a worthy implant removal, whereas cosmetical aspects were unsuitable. Preconditions for satisfaction of over 90% after implant removal were identified, including a thorough assessment of the indication factors, standardized approach, avoidance of wound enlargements, and possibly a mobilization of the shoulder under anesthesia after implant removal.

## Disclaimers:

Funding: No funding was disclosed by the authors.

Conflicts of interest: The authors, their immediate families, and any research foundation with which they are affiliated have not received any financial payments or other benefits from any commercial entity related to the subject of this article.
